# Occupational risk perceived by pregnant workers: proposal for an assessment tool for health professionals

**DOI:** 10.47626/1679-4435-2020-550

**Published:** 2020-12-11

**Authors:** Nathália Beatriz Manara Lellis, Valmir Azevedo, Sergio Roberto de Lucca, Marcelo Pustiglione, Marcia Cristina Bandini

**Affiliations:** 1 Medicina do Trabalho, Universidade Estadual de Campinas - Campinas (SP), Brazil; 2 Centro de Referência de Saúde do Trabalhador (Cerest), Centro de Vigilância Sanitária, Secretaria de Estado da Saúde de São Paulo - São Paulo (SP), Brazil

**Keywords:** pregnancy, occupational risks, occupational exposure, risk assessment, occupational medicine

## Abstract

**Introduction:**

The risk factors and agents present in the work environment may represent a risk to the health of pregnant women, the developing infants, and breast-feeding mothers; however, tools to assess occupational exposure of these workers are not available.

**Objective:**

To develop an instrument for the qualitative assessment of occupational exposure of pregnant workers based on their perceptions.

**Method:**

We conducted a data survey from the National Institute for Occupational Safety and Health and the Brazilian Regulatory Standard 15. Next, a comparative analysis was performed, according to the scientific literature available, followed by a preliminary version of the instrument, a pilot test with 15 pregnant women, and preparation of the final version.

**Results:**

A tool was developed consisting of 28 questions, divided into 7 categories: 1) pregnant or lactating woman; 2) habits and behaviors; 3) information about work; 4) risk factors identified by the worker in the work environment divided into chemical, physical, biological, ergonomic, and accidents; 5) difficulties faced at work; 6) need for antenatal leave; and 7) open question so that the worker can inform something she considers necessary.

**Conclusions:**

The study of work-related risk factors and/or agents relevant to the health of pregnant women and/or the fetus is essential to conduct adequate prenatal care and to protect the health of these workers. The use of this tool can be of great value for health professionals, especially for physicians. The practical application can bring possible improvements that were not identified by the authors during the study.

## INTRODUCTION

Women’s participation in the labor force has been increasingly growing in Brazil, especially in recent decades. According to the demographic census of the Brazilian Institute of Geography and Statistics (IBGE), in 1950, women represented 13.6% of the economically active population, and in 2010, this participation jumped to 49.9%.^1^ Also according to the IBGE, in 2010 women already represented around 43.5% of the economically active population.^2^

Several studies show that numerous risk factors and agents considered harmful to health may be present in the work environment, representing a potential risk for the pregnant woman, the developing infant, and the nursing mother. As an example of chemical agents, we can mention anesthetic gases and formaldehyde. Anesthetic gases are associated with an increased risk of miscarriage,^3^ and formaldehyde is known for its oncogenic characteristics, can lead to infertility, miscarriage, and is excreted by breast milk during breastfeeding.^4^^,^^5^

Concerning physical agents, heat can lead to reproductive problems, fetal malformations, and placental detachment in term pregnancies, according to the National Institute for Occupational Safety and Health (NIOSH). Noise, which is one of the most common risk factors in different production processes, can induce hearing problems in the mother and also in the fetus, besides the fact that occupational exposures above 85 dB during pregnancy (full-time workers) were associated with a higher risk of restricted intrauterine growth.^3^^,^^6^ An example of biological agent is the Zika virus, whose infection during pregnancy can cause microcephaly and severe brain malformations in the fetus. In addition to the risk factors and agents mentioned, it is also possible to find an association of effects on the health of pregnant workers with ergonomic factors, night work, and long hours of work.^3^

Therefore, there are many risk factors and agents present in the workplace and, consequently, an extensive amount of scientific evidence in the national and international literature on the relationship of exposure to such factors/agents and the health of the pregnant/breastfeeding woman in the workplace. The recognition of these risks and the adoption of the appropriate conduct are highly necessary and even intrinsic to the routine of the obstetrician and the occupational physician to protect the health of the worker and the infant.

However, health professionals are not always prepared to identify such risk factors or agents, including the obstetrician or the occupational physician. The literature lacks tools to facilitate the assessment of occupational exposure and the impact of this exposure on the life of pregnant workers. In a recent literature review, Pustiglione^7^ suggests a model of occupational risk assessment (ORA), which aims to be a theoretical reference, with information on risk factors and agents for pregnant and breastfeeding women and the consequent impacts on the fetus and infant, guiding decision-making in both theoretical and legal aspects.

Even when the ORA is conducted, there is still the difficulty of making the best decision which, on the one hand, preserves the health of the pregnant woman and the fetus and, on the other hand, maintains the pregnant woman’s right to work, avoiding possible discrimination related to pregnancy. This challenge is routinely presented to professionals, especially members of the family health teams, the obstetrician, and, mainly, the occupational physician.

In addition to the complexity of the subject from a clinical and toxicological perspective, the challenge is even greater in face of recent changes in Brazilian legislation. Until 2016, there was no clarity on the theme of the work of pregnant women in hazardous conditions. On May 11, 2016, the Law No. 13.287 was sanctioned, which added Article 394-A to the Brazilian Consolidation of Labor Laws (CLT). As a result of this provision, this law came into force with the addition of the aforementioned article, which textually states that: “the pregnant or nursing employee shall be kept away from any activity, operation or unhealthy place during pregnancy and lactation, and shall perform her activities in a salubrious place.”^8^ On July 13, 2017, a change was sanctioned in the wording of article Art. 394-A: “Without prejudice to their remuneration, including the amount of the hazard pay, the employee must be removed from: I - activities considered hazardous in a greater degree, during pregnancy; II -activities considered hazardous in medium or minimum degree, when presenting a health certificate, issued by a physician trusted by the woman, who recommends work leave during pregnancy; III -activities considered hazardous in any degree, when presenting a health certificate, issued by a doctor trusted by the woman, who recommends the work leave during lactation.”^8^ Law No. 13.467 further establishes, in its Paragraph 3, that: “When it is not possible for the pregnant or nursing woman who is away under the caput of this article to conduct her activities in a healthy place in the company, the hypothesis will be considered as a risk pregnancy and will give rise to the right to receive maternity wages, under the terms of Law No. 8.213, of July 24, 1991, during the entire period of absence.” However, on May 29, 2019, the Brazilian Federal Supreme Court (STF), by majority of votes, upheld the Direct Action of Unconstitutionality (ADI) No. 5.938, to declare unconstitutional the sections of the CLT provisions inserted by the Labor Reform (Law No. 13.467, of July 13, 2017), which admitted the possibility of pregnant and breastfeeding workers to perform hazardous activities in some cases.^9^ For the majority, the expression “when presenting a health certificate, issued by a doctor trusted by the woman,” contained in clauses II and III of the article 394-A of the CLT, violates the constitutional protection of maternity and children. As a result, Law No. 13.287, of May 11 2016 -the original law - came into force again.

In this context, it is necessary that health professionals are competent to assess risk factors and agents related to the work of pregnant and nursing women and that they can support the clinical and occupational anamnesis, guiding a qualified listening to the perception of the worker herself, regardless of her employment relationship, about her working conditions, environments, and processes.

Thus, the present study aimed to develop an instrument, in Brazilian Portuguese, that helps health professionals, especially physicians, to assess occupational exposure to risk factors and agents of interest, from the perception of the pregnant worker, to establish the conduct to be adopted and the need for guidance, when relevant, to the patient and the employer on possible measures of control and protection.

## METHOD

A questionnaire was developed by the Universidade Estadual de Campinas (UNICAMP), in a process composed of 5 phases:

1. Survey of risk factors/agents based on the recommendations of the Centers for Disease Control and Prevention (CDC)/NIOSH published in Reproductive Health and the Workplace, focusing on the sector Specific Exposures during Pregnancy and Breastfeeding, which provides information for employers and workers.
2. Survey of risk factors /agents present in Regulatory Standard-15 (RS-15), from its annexes.
3. Comparison of the criteria identified in phases 1 and 2, with the consolidation of risk factors and/or agents with potential risk of producing health problems for pregnant and breastfeeding workers, according to the available scientific literature. Preparation of the preliminary version of the instrument.
4. Preliminary version test with 15 pregnant women interviewed in an outpatient clinic specialized in high-risk pregnancies, after free and informed consent.
5. Preparation of the final version.

## RESULTS

In RS-15, 15 risk factors were identified for the purpose of paying the hazard pay, regardless of the pregnancy situation. In the survey of the criteria considered in the recommendations of the CDC / NIOSH, specific for pregnant and breastfeeding women, 17 risk factors and/or agents were found. The data were compared with each other and there was no direct correlation between the references, which hindered the comparison by risk factor/agent. For example, while CDC / NIOSH brings the “solvent” agent in a generic way, the RS-15 describes some solvents for the purpose of characterizing hazard. Even so, almost all of the criteria used by RS-15 are also recognized as potentially hazardous by the CDC / NIOSH recommendations. The exception was “humidity”, which is recognized in Brazil for additional medium-grade hazard and does not appear in the CDC/NIOSH recommendations. The opposite is true for pesticides that are not described in RS-15, but are described by CDC / NIOSH. A summary of the findings is described in Table 1, which served as a basis for the preparation of the first version of the instrument.

**Table 1:** Comparison between risk factors and agents of the Centers for Disease Control and Prevention/National Institute for Occupational Safety and Health (CDC/NIOSH) and Regulatory Standard 15 (RS-15) according to their inclusion in the research instrument

	NIOSH	RS-15	Research instrument
Physical risk			
Noise	+	+	+
Heat	+	+	+
Vibration (WBV/HAV)	+	+	+
Cold	+	+	+
Hyperbaric conditions	+	+	+
Humidity		+	
Ionizing radiation	+	+	+
Non-ionizing radiation	+	+	+
Chemical risk			
Anesthetic gases	+	+	+
Antineoplastic drugs	+		+
Formaldehyde	+	+	+
Pesticides	+		+
Epoxies and resins	+	+	+
Disinfectants and sterilizers	+	+	+
Heavy metals	+	+	+
Smoke from by-products of burning	+	+	+
Solvents	+	+	+
Dust	+	+	+
Biological agents	+	+	+
Ergonomic risk			
Physical demand	+		+
Night shift work	+		+
Long working hours	+		+
Accident risk	+		+

HAV: hand and arm vibration; WBV: whole body vibration.

From the comparative analysis, a preliminary version of the instrument was developed, composed of 28 questions, divided into 7 categories: 1) data on the pregnant/breastfeeding woman; 2) habits and behaviors; 3) information on the work; 4) risk factors identified by the worker in the work environment, divided into chemical, physical, biological, ergonomic, and risk of accidents; 5) difficulties faced at work; 6) need for a work leave; and 7) open question so that the worker can inform something she deems necessary.

This preliminary version was tested with 15 pregnant women from a convenience sample among patients at the university’s high-risk pregnancy outpatient clinic. Pregnant women were informed of the purpose of the test and agreed to participate in the interviews with the researcher. The questionnaire was administered by interview, lasting approximately 10-15 minutes. After the test, few adjustments were necessary to prepare the final version. Questions about the period during which the pregnant woman worked on a particular activity were included as well as a final open question to add any comments, if the interviewee considered it relevant. The list of risk factors and work-related agents was not changed. Thus, the final version of the instrument in Brazilian Portuguese was completed, presented in a supplementary online file.

## DISCUSSION

There are many agents and risk factors intrinsic to work that are consequently present in a considerable part of the workers’ routine, some of which offer potential health risks. In the case of pregnant workers, there is still the risk of affecting not only their own health, but also that of the fetus. Thus, the importance of adequate recognition of occupational exposure for all workers, regardless of formal or non-formal employment, is emphasized, especially for workers in situations of greater vulnerability or susceptibility. Considering the perception of workers regarding the risks to which they are exposed in workplaces or environments is essential for all medical professionals.

The application of the pilot instrument showed that pregnant women have a good perception of the risks to which they are exposed. Therefore, the use of a tool that helps to identify the risk factors and agents present in the work environment can be of prenatal relevance, reflecting the possible risks to the health of the worker and the developing infant. Moreover, the application of the pilot tool proved to be feasible in practice due to the short administration time of approximately 10 to 15 minutes.

Most of the risk factors and agents commonly found have studies in the literature that show and corroborate the potential hazard during pregnancy. Reid et al.^10^ described the possible relationship between exposure to asbestos dust and the occurrence of choriocarcinoma, a disease also known as hydatidiform mole. The authors identified this association both in women exposed directly and in those who lived with workers from an asbestos company. Asbestos fibers in the lung, in the pleural and peritoneal mesothelium, and the ovaries were detected in the women participating in the study and in the placenta and digestive tract of live and stillborn neonates.^10^

In the health area, where the workforce is predominantly comprised of women, female workers are exposed to various risk factors. A meta-analysis investigated the exposure of nurses to anesthetic gases in operating rooms and showed a significant risk of miscarriage.^11^ Anderson and Goldman^12^ published a review that demonstrated the occurrence of numerous occupational risks in a surgical center that can influence adverse pregnancy outcomes and increase the infertility rate. According to Haffner et al.,^13^ the results of most studies of pregnant women in contact with formaldehyde suggest that, by avoiding contact during pregnancy, there may be a reduction in the relative risk of low birth weight, miscarriage, and malformations.^13^

Pesticides are an example of a risk factor found in NIOSH, with considerable evidence in the literature, both in older studies and recent ones, but absent in the RS-15. A study conducted between 1996 and 2000 in southern Brazil showed a possible relationship between the use of pesticides and reproductive effects, such as preterm birth.^14^ Wright et al.^15^ conducted a prospective cohort study that assessed occupational exposure to endocrine-disrupting chemicals during pregnancy, showing that maternal occupational exposure to pesticides is associated with impaired intrauterine fetal growth.

As for solvents, they are known to be associated with a possible decrease in fertility, risk of congenital malformation, and miscarriage. Vaktskjold et al.^16^ found that pregnant women exposed to organic solvents, especially painters, present a greater risk of having a baby that is small for gestational age. The study also shows that the average weight of a child born to a mother exposed to organic solvents was 2185 g lower than the mean weight of those not exposed [95% confidence interval (95% CI)] and statistically lower even between term newborns and of the same gestational age.

The noise, found in several work environments, has its action on the human body well understood with the activation of the pituitary-adrenal-cortical axis and the sympathetic-adrenal-medullary axis, as well as the stimulation of stress hormones, including epinephrine, norepinephrine, and cortisol.^17^ Babisch^18^ verified in a review that, in addition to the changes already known in the nervous and endocrine systems, there is an increase in concentrations of these hormones in the blood, being an important stress factor. Gélat et al.,^19^ as well as previous authors, conducted a study with sheep models due to the similarity of a human term uterus with the sheep uterus. As a conclusion, they corroborated previous studies that showed epidemiological evidence that pregnant women should not be exposed to high occupational noise in the long term. Selander et al.^20^ demonstrated the relationship between exposure to high levels of sound pressure (> 85 dB) during pregnancy and an increased risk of low-birth-weight neonates. In addition, it was also found that exposure to noise acts through the hypothalamic-pituitary-adrenal axis, stimulating increased secretion of stress hormones, with the consequent increase in blood pressure and heart rate in pregnant women.^20^

Perhaps ionizing radiation is the risk with the best-known effects and, therefore, avoided. It is known that it can lead to several changes in pregnancy, including fetal death, mutagenesis, and carcinogenesis.^21^

There are several infectious agents capable of causing changes in the fetus and, among them, Rubivirus, which causes the congenital rubella syndrome, and cytomegalovirus, which leads to birth and developmental defects.^22^^,^^23^ According to Morales-Suárez-Varela et al.,^24^ women who have contact with patients tend to experience miscarriage and a higher prevalence of having an infant born with congenital malformation.^24^

As for ergonomics and accident risk, also absent in RS-15, there are some effects found in the literature.

The practice of high intensity exercises, for example, creates a state of hypoxia for the fetus, which can lead to intrauterine growth restriction and prematurity.^25^ In the case of abdominal trauma, there is a risk of placental abruption, prematurity, and even fetal death, especially with advancing pregnancy.^26^

**Figure 1:**
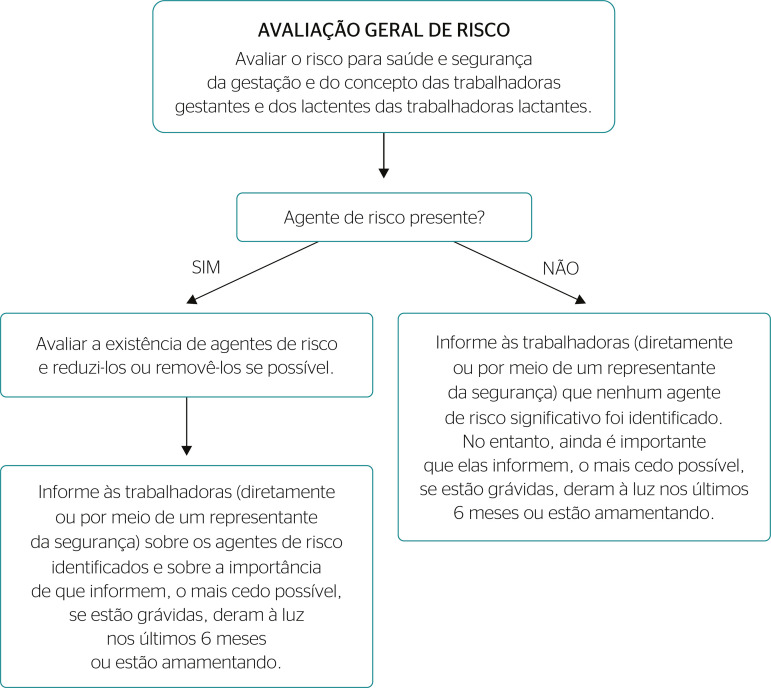
Flowchart of general risk assessment

In a recent literature review, Pustiglione^5^ proposes an algorithm for the ORA facilitate decision-making by medical professionals in the case of exposure with the potential to produce complications and/or diseases to the pregnant woman and the fetus (Figure 1). However, the studies identified the need to develop an instrument that would use national and international criteria for this evaluation.

The proposed instrument can be used and must be improved through its application in clinical practice, either during the prenatal care of obstetricians and gynecologists, during the periodic examinations performed by occupational physicians or in the monitoring of family and community physicians, among other professionals who have pregnant workers as patients.

## CONCLUSION

The study of the risk factors and agents present in the work environments, relevant to the health of the pregnant woman and/or the developing infant, is essential to conduct adequate prenatal care and to protect the health of these workers. The use of an instrument for the recognition of occupational risks in the working environment of pregnant women can be of great value for health professionals, especially physicians. The practical use of the instrument can bring possible improvements that were not identified by the authors during the application of the pilot test.
